# Anxiolytic Activity of Morellic Acid: Modulation of Diazepam's Anxiolytic Effects, Possibly Through GABAergic Interventions

**DOI:** 10.1111/cns.70276

**Published:** 2025-02-17

**Authors:** Md. Shimul Bhuia, Tanzila Akter Eity, Raihan Chowdhury, Siddique Akber Ansari, Mehedi Hasan Bappi, Md. Anin Nayeem, Farjana Akter, Muhammad Torequl Islam

**Affiliations:** ^1^ Department of Pharmacy Bangabandhu Sheikh Mujibur Rahman Science and Technology University Dhaka Bangladesh; ^2^ Bioinformatics and Drug Innovation Laboratory BioLuster Research Center Ltd. Dhaka Bangladesh; ^3^ Department of Biotechnology and Genetic Engineering Bangabandhu Sheikh Mujibur Rahman Science and Technology University Dhaka Bangladesh; ^4^ Department of Pharmaceutical Chemistry, College of Pharmacy King Saud University Riyadh Saudi Arabia; ^5^ School of Pharmacy Jeonbuk National University Jeonju Republic of Korea; ^6^ State University of Bangladesh Dhaka Bangladesh; ^7^ Pharmacy Discipline Khulna University Khulna Bangladesh

**Keywords:** anxiety, GABAergic transmission, molecular docking, morellic acid, neuroprotective, xanthones 3

## Abstract

**Background:**

Numerous studies suggest that morellic acid (MOR), highly available in Garcinia plants, has different physiological activities, including anti‐cancer, anti‐oxidant, and anti‐microbial activity.

**Aim:**

In this investigation, we aimed to demonstrate the anxiolytic activity, along with the mechanism behind this activity of MOR, using in vivo and *in silico* studies.

**Methods:**

For this, we used different doses of MOR (5 and 10 mg/kg) and administered this drug intraperitoneally to *Swiss* albino mice (male and female). Diazepam (DZP), a positive allosteric modulator of the GABA_A_ receptor, was used as a positive control at a dose of 2 mg/kg (i.p), and vehicle was used as a control group. In this test, various test protocols are used to assess the behavioral patterns of mice, including swing, hole cross, light–dark testing, and open field testing.

**Results:**

This investigation revealed that MOR remarkably reduced the locomotor activity of mice in a dose‐dependent manner and produced calming behaviors like DZP. However, the findings showed that the combination of MOR and DZP synergistically reduced the locomotion of mice compared to the single therapy. On the other hand, from the computational study, the result demonstrated that MOR exhibited the highest binding scores (−9.2 kcal/mol) towards the GABA_A_ receptor α3 subunit and −7.6 kcal/mol towards the GABA_A_ α2 receptor. Whereas, DZP showed −6.6 and −7.3 kcal/mol docking affinity and FLU exerted −6.2 and −6.3 kcal/mol docking scores towards the GABA_A_ receptor α2 and α3 subunits, respectively. The ligand interacted with the receptor by forming different hydrogen and hydrophobic bonds.

**Conclusion:**

However, it is recommended that more precise and comprehensive preclinical investigations be required to demonstrate the exact mechanism behind the anxiolytic effects and conduct clinical trials to determine efficacy and safety.

## Graphical abstract

Morellic acid (MOR) alone, dose‐dependently, and with the standard drug diazepam (DZP), significantly (*p* < 0.05) reduced the locomotive activities of Swiss mice. Additionally, the computational studies suggest that MOR exhibited the highest binding scores of −9.2 and −7.6 kcal/mol towards the GABA_A_ receptor α_3_ and α_2_ subunits, respectively.

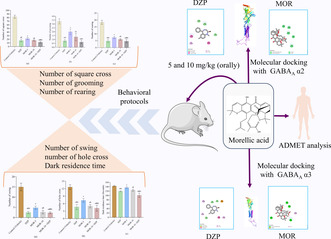

## 1 Introduction

The most common psychiatric disorder, anxiety, is characterized by an uncomfortable feeling of vague fear or apprehension in humans. Anxiety affects approximately 25% of the world population at least once in their lifetime [1, 2]. Based on the observation of the World Health Organization (WHO), anxiety affects around 260 million people globally [3]. Current evidence suggests that generalized anxiety disorder is the most common anxiety disorder among the six main types of anxiety disorders [4, 5]. It should be clear that anxiety is an adaptive mechanism that is necessary for the organism's survival. However, in extreme conditions, individuals develop discrete disorders that are commonly comorbid with other psychological disturbances [6, 7]. Benzodiazepines (BZDs) (e.g., diazepam, lorazepam, midazolam, and alprazolam) are commonly used in anxiety [8], although these drugs in the long run are evident to decrease their efficacy in anxiety patients, produce mild to serious side effects, and are highly misused [9]. Moreover, these drugs result in incomplete remission in most patients [10]. Therefore, it is an important issue to search for new, effective, and safer anxiolytic drugs from various sources.

Anxiety decreases brain GABA levels in animals [11]. GABA_A_ is a complex receptor that is comprised of various subunits, such as *α*, *β*, and *γ* [12]. BDZs (including diazepam) are positive allosteric modulators of the GABA_A_ receptor, and they are ion channels. The ion channels are ligand‐gated chloride‐selective and are triggered by GABA. Therefore, an increased brain GABA level increases the chloride ion influx inside the neuronal cells and causes hyperpolarization of the neuron's membrane potential. As a result, a calming effect occurs on the central nervous system (CNS). Diazepam (DZP) is commonly used for anxiety, insomnia, panic attacks, acute alcohol withdrawal, sedation, amnesia, and epilepsy [13]. However, repeated administration of DZP results in tolerance in animals [14].

Plants and their derivatives, commonly recognized as one of the potential classes of natural products, have been identified as potential targets for anxiety [15, 16]. Morellic acid (MOR: xanthones 3), mainly found in Garcinia plants, is evident for its anticancer properties [17]. It also has different pharmacological properties, including anti‐inflammatory, antioxidant, antimicrobial, anti‐obesity, antivirals, and anti‐diabetic [17, 18, 19, 20]. A prior study indicated that xanthones (MOR) exhibit neuroprotective properties by enhancing cell survival and diminishing the buildup of β‐amyloid and tau aggregation [21]. However, the therapeutic interventions regarding its anxiolytic effect are yet to be discovered. Thus, the current study aimed to assess the anxiolytic effect of MOR in *Swiss* albino mice. Likewise, we combined MOR with a GABA‐agonist drug called diazepam for the identification of synergistic or antagonistic properties. Additionally, we also carried out *in silico* studies with the GABA_A_ receptor to understand the possible anxiolytic effects of the test sample and the standard drugs.

## 2 Materials and Methods

### 2.1 Reagents and Chemicals

Morellic acid (MOR) was purchased from Sigma Aldrich (St. Louis, MO, USA) (CAS: 5304‐71‐2, purity: ≥ 95% HPLC), while diazepam (DZP) and flumazenil (FLU) were kindly supplied by Square Pharmaceuticals Ltd. (Bangladesh). Tween 80 required for this study was purchased from Merck (India).

### 2.2 Experimental Animals

Adult male and female *Mus musculus* (*Swiss* albino mice; avg. b.w. 26–30 g, age: 6–8 weeks) gathered from the Animal House of Jahangirnagar University, Bangladesh, were randomly distributed into different groups (*N* = 42; *n* = 6). Before that, the animals were housed in an optimal environment (temperature: 25°C ± 2°C, relative humidity: 65%) for 7 days in several rectangular housing boxes (290 × 220 × 140 mm). We kept 5 mice per box. They had free access to standard foods and water *ad libitum*. Studies were performed between 9:00 am and 3:00 pm. Animals involved in this experiment were fasted overnight. However, they were allowed free access to water only. This study was approved by the Animal Ethics Committee of Khulna University (KUAEC‐2023‐05‐09).

### 2.3 In Vivo Study

#### 2.3.1 Groups and Treatments

The test doses for this study of MOR (5 and 10 mg/kg, i.p) were selected from the previously existing literature [22] and based on acute toxicity tests. DZP was administered at 2 mg/kg, while the control (Vehicle: distilled water containing 0.9% NaCl and 0.5% tween 80) was given at 10 mL/kg. Besides, FLU is administered at 0.1 mg/kg (i.p). A combination group was comprised of MOR and DZP at 10 and 2 mg/kg, respective doses (Table 1). In this case, we followed one after another administration to the animals. Before treatment, all animals were fasted overnight. The mice were randomly categorized into five groups, each comprising five animals. They were treated intraperitoneally (i.p) 30 min before starting the study and in a single set of animals. Then, follow the below‐mentioned studies. All the behavioral data were collected by the close stay of an investigator manually.

**TABLE 1:** Groups and treatments via intraperitoneal administration.

Treatment Groups		Description	Dose	Administration design
*Individual groups*	Control (Vehicle)	Distilled water containing 0.9% NaCl and 0.5% tween 80	10 mL/kg	At a time (i.p)
	DZP‐2	Diazepam [agonist]	2 mg/kg	At a time (i.p)
	FLU‐0.10	Flumazenil [antagonist]	0.10 mg/kg	At a time (i.p)
	MOR‐5	Morellic acid	5 mg/kg	At a time (i.p)
	MOR‐10	Morellic acid	10 mg/kg	At a time (i.p)
*Combination group*	MOR‐10 + DZP‐2	Morellic acid+Diazepam	10 + 2 mg/kg	One followed by another (i.p)
	MOR‐10 + FLU‐0.10	Morellic acid+ Flumazenil	10 + 2 mg/kg	One followed by another (i.p)

*Note:* *N* = 42; *n* = 6.

Abbreviations: DZP, Diazepam; FLU, Flumazenil; i.p, intraperitoneal; MOR, Morellic acid.

#### 2.3.2 Open‐Field Test

The experiment was conducted using the methodology outlined by Tan et al. [23], with several adjustments made. To assess the spontaneous movement (NSC), grooming (NG), and rearing (NR) behaviors of the animals, a stainless steel (SS) field measuring 20 × 16 in. was utilized. The SS field was bordered by a wooden wall measuring 9 in. in height on all four sides. This test was conducted for 5 min [23]. The field contains 16 equal squares that were properly cleaned with 65% ethanol after the performance of each animal.

#### 2.3.3 Hole‐Cross Test

This study was done according to de Almeida et al. [24]. Briefly, a wooden box (30 × 20 × 14 cm^3^) containing a hole at the center of the box was used. The hole's diameter was 3 cm, and it was drilled in the lowest part of the dividing board of the box placed in the middle of it. This test was followed by the previous study (open‐field test) for 5 min after a 1 min resting phase. In this case, the number of spontaneous movements of each animal was recorded through the hole (NHC) [24]. The floor of the box was also cleaned using the same procedure as mentioned above.

#### 2.3.4 Swing Test

The experimental animals' swing behavior was assessed using the approach described by Islam et al. [25]. This test was conducted after the hole‐cross study, followed by a 1 min resting period. Besides, this test was performed for 5 min. Each animal was placed at the center of the swing box and counted the number of swings (NS) within the observation time [25]. The floor of the apparatus was also cleaned as said before.

#### 2.3.5 Dark–Light Test

**FIGURE 1:**
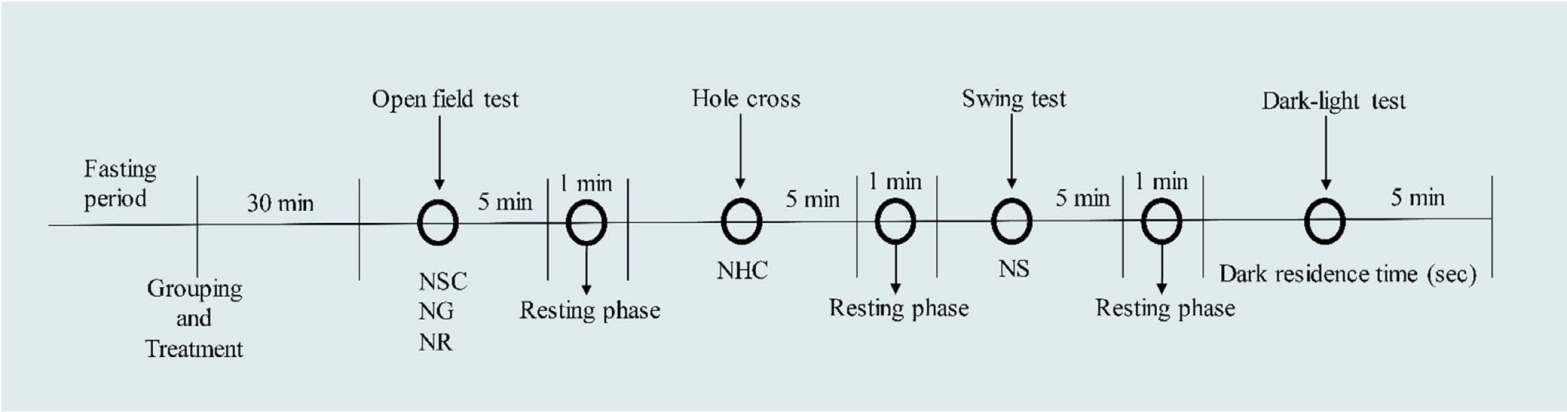
Study outline for anxiolytic test in *Swiss* albino mice.

This study is a modification of Subhan et al. [26]. It contains a box with equal measurements (27 × 18 × 29 cm^3^) of light and dark parts. The illumination of the light portion is derived from the surrounding ambient light, and the intensity of light was ~300 lx, whereas the dark portion is entirely obscured by a 3‐cm diameter aperture located in the lower section of the dividing board [27]. After the swing test, each animal was placed in the light box of this apparatus for 5 min and observed for the dark‐residence time (DRT) (in seconds) using a stopwatch [26]. The floor of the equipment was also cleaned as stated before (Figure 1).

#### 2.3.6 Statistical Analysis

Values were expressed as mean ± SEM (standard error of the mean). One‐way ANOVA followed by Student–Newman–Keuls post hoc test with multiple comparisons was performed at 95% confidence intervals using GraphPad Prism software (version 9.5, San Diego, USA). Data were considered statistically significant when *p* < 0.05.

### 2.4 In Silico Study

#### 2.4.1 Homology Modeling and Macromolecules Preparation

Our investigation centered on the GABA_A_ receptor α2 and α3 subunits, recognized for their implications in anxiety according to extant literature. However, due to the unavailability of the three‐dimensional (3D) structures of these subunits (α2 and α3 subunits) within the human GABA_A_ receptor in the RCSB Protein Data Bank, we employed homology modeling techniques. We utilized the SWISS‐MODEL platform to construct homology models for the α2 and α3 subunits of the human GABA_A_ receptor. To ensure fidelity and appropriate template selection, we retrieved the protein sequences corresponding to the α2 and α3 subunits (UniProt ID: P47869 and P34903, respectively) from the UniProt database. Subsequently, we conducted a BLAST assessment using the NCBI BLAST tool to identify suitable templates. We evaluated the quality of the resultant homology models using metrics such as GMQE and through the evaluation of Ramachandran plots via ProCheck. Following model validation, we undertook optimization procedures to mitigate potential perturbations during molecular docking. We systematically removed extraneous elements such as lipids, water molecules, and heteroatoms from the protein sequences using PyMol software (version 2.4.1). Following this, energy minimization and geometry optimization of the receptors were performed using SwissPDB Viewer software with the GROMOS96 force field [28]. We saved the resultant optimized structures in PDB format to facilitate subsequent molecular docking analyses.

#### 2.4.2 Ligands Preparation

**FIGURE 2:**
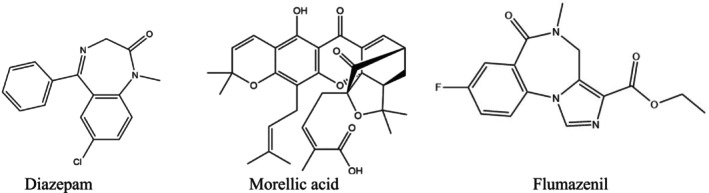
The chemical structures of diazepam, flumazenil, and morellic acid.

The selected chemical compounds of MOR (Compound CID 54580250) and DZP (Compound CID 3016) were collected from the PubChem database (https://pubchem.ncbi.nlm.nih.gov; accessed on 9 June 2024) in 3D structure and stored in SDF format. Afterward, for the minimization of energy in these compounds, Chem3D 16.0 software was used [29]. Then, for the molecular docking approach, the minimized compounds were saved in SDF format. The 2D chemical conformers of the compounds are illustrated in Figure 2.

#### 2.4.3 Molecular Docking and Visualizations

We performed molecular docking of MOR and DZP with the GABA_A_ receptor α2 and α3 subunits using the PyRx software package. This procedure was employed to predict the active binding potential of the drugs at the receptor's active sites. To initiate the docking process, we enclosed the receptor and ligand within a grid box, setting the dimensions to maximum values along the x‐, y‐, and z‐axes, and executed the calculation over 200 steps. The docking results were saved in CSV format, and we extracted the best pose, determined by ligand binding affinity and RMSD lower and upper bound values, in PDB format for further analysis. Using Discovery Studio Visualizer (v21.1.020298), we analyzed the interactions between the ligand and receptor at the active site. We documented the amino acid residues involved, bond types, hydrogen bond lengths, and other interaction details for each ligand‐receptor complex [30, 31].

#### 2.4.4 Pharmacokinetic and Drug‐Likeness Properties

The assessment of ADMET features, which examine the body's reaction to drugs as they are metabolized and removed over time, is the main goal of statistical pharmacokinetics studies. The *in silico* method transforms a chemical into a therapeutically useful medication by analyzing its early pharmacokinetic properties [32]. In the field of research and development, “drug‐likeness” is a qualitative evaluation that gauges how much a chemical molecule mimics a therapy under specific circumstances. The drug‐likeness and ADME properties of MOR were evaluated using the SwissADME database [33].

#### 2.4.5 Toxicity Analysis

The ProTox 3.0 web server was employed to evaluate the toxicity properties of SES. For this, we collected the SMILES from PubChem and input them in the ProTox 3.0 search box [34]. The different toxicity parameters were analyzed, and the designated properties documented.

## 3 Results

### 3.1 In Vivo Findings

In the acute toxicity test, MOR did not show any toxicity or abnormalities during our 7‐day observation period on 5, 10, 20, 50, 100, and 200 mg/kg doses in experimental animals. In this case, we followed four well‐established test protocols as mentioned above. The findings of these test modalities are shown below.

#### 3.1.1 Open‐Field Test

**FIGURE 3:**
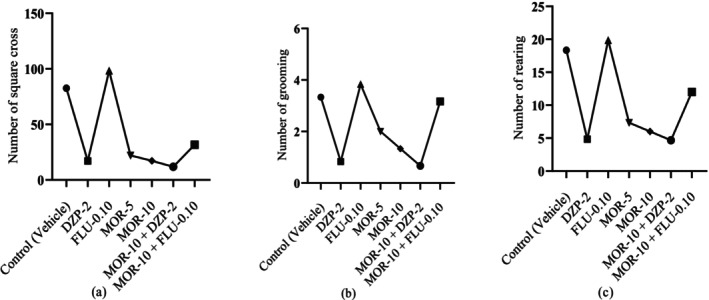
Behavioral observations across test and control groups: (a) Number of square crosses (NSC), (b) number of grooming (NG), and (c) Number of rearing (NR). [Values are presented as mean ± standard error of the mean (SEM). Statistical analysis was performed using one‐way ANOVA followed by Student–Newman–Keuls post hoc test with multiple comparisons at 95% confidence intervals; Degree of freedom (DF): 35; *p* < 0.05 when *compared to the control (vehicle) group; ^a^compared to the DZP‐2; ^b^compared to the FLU‐0.10; ^c^compared to the MOR‐5; ^d^compared to the MOR‐10; DZP‐2: Diazepam (2 mg/kg); FLU‐0.10: Flumazenil (0.10 mg/kg); MOR‐5 and 10: Morellic acid (5 and 10 mg/kg)].

According to Figure 3, the standard drug DZP showed an NSC value of 17.00 ± 3.70, which was significant (*p* < 0.05) in comparison to the control group's NSC value (82.50 ± 4.30). The test drug MOR dose‐dependently reduced the NSC in animals, where at 5 mg/kg (22.00 ± 3.25) and at 10 mg/kg (17.17 ± 2.02) it produced a DZP‐like effect in animals. MOR at 10 mg/kg when combined with DZP at 2 mg/kg resulted in the best reduction of NSC value in animals (11.83 ± 2.33). Similarly, the DZP also significantly (*p* < 0.05) reduced NR (4.83 ± 1.05) and NG (0.83 ± 0.17) values in animals compared to the control group (NR: 18.33 ± 1.48; NG: 3.33 ± 0.33). MOR also dose‐dependently and significantly (*p* < 0.05) reduced these parameters in comparison to the control group. Its highest dose (10 mg/kg) reduced these values more proficiently than its 5 mg/kg dose. The combination group MOR‐10 + DZP‐2 exhibited the best NR and NG reduction capacity than the individually treated groups by DZP‐2 and MOR‐10. Moreover, the combination group of FLU and MOR exhibited reduced NSC (31.67 ± 5.29) and NG (3.16 ± 0.40) as well as increased NR (19.83 ± 1.40) compared to FLU‐0.10 group (NSC: 98.17 ± 3.34; NR: 12 ± 1.41; NG: 3.83 ± 0.40). The MOR‐10 group, subjected to combined treatment with DZP and FLU, exerted remarkable alterations in all test measures in comparison to the control, MOR‐10, and FLU‐0.10 groups. However, the FLU‐0.10 showed a lower Cohen's D (Effect size) value compared to the control. The effect size in the open‐field test of all treatment groups is provided in Table S1.

#### 3.1.2 Swing Test

**FIGURE 4:**
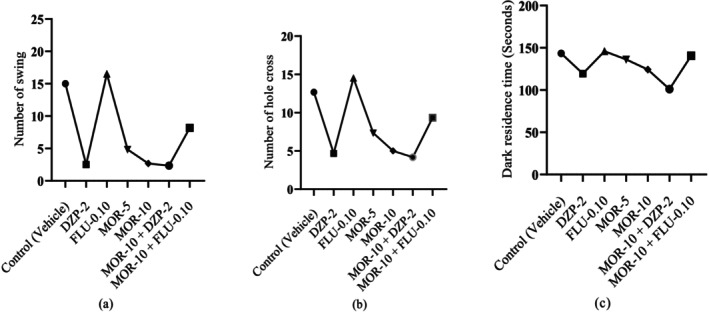
Behavioral observations across test and control groups: (a) Number of swing (NS), (b) number of hole cross (NHC), (c) dark residence time (DRT) [Values are presented as mean ± standard error of the mean (SEM). Statistical analysis was performed using one‐way ANOVA followed by Student–Newman–Keuls post hoc test with multiple comparisons at 95% confidence intervals; Degree of freedom (DF): 35; *p* < 0.05 when *compared to the control (vehicle) group; ^a^compared to the DZP‐2; ^b^compared to the FLU‐0.10; ^c^compared to the MOR‐5; ^d^compared to the MOR‐10; DZP‐2: Diazepam (2 mg/kg); FLU‐0.10: Flumazenil (0.10 mg/kg); MOR‐5 and 10: Morellic acid (5 and 10 mg/kg)].

Figure 4 suggests that the reference drug DZP significantly (*p* < 0.05) reduced NS value (2.50 ± 0.56) in comparison to the control group (15.00 ± 1.86). However, FLU exerted the highest NS (16.50 ± 2.11) compared to all groups. MOR also dose‐dependently and significantly (*p* < 0.05) reduced NS value in comparison to the control group, where at 10 mg/kg (2.67 ± 0.84) it produced a DZP‐*like* NS value in animals, and at 5 mg/kg, it showed 4.83 ± 0.79 NS value. The combination group (MOR+DZP‐2) showed the lowest NS value (2.33 ± 0.42) in animals. Besides, the combination of MOR‐10 and FLU‐0.10 exhibited a higher NC value (8.17 ± 0.95).

#### 3.1.3 Hole‐Cross Test

The control group exhibited NHC of 12.67 ± 0.95, which was significantly (*p* < 0.05) reduced by the standard drug DZP (4.67 ± 1.05). The animals that were given FLU treatment showed the highest NHC (14.50 ± 1.05) compared to all other groups. MOR dose‐dependently and significantly (*p* < 0.05) reduced NHC in comparison to the control group animals, where MOR‐5 showed NHC value of 7.33 ± 0.76 and MOR‐10 exhibited 5.00 ± 0.68, which produced a DZP‐like effect in animals. The combination group of MOR‐10 and FLU‐0.10 exhibited higher NHC (9.33 ± 1.33) than the DZP group. However, In the combination group, MOR‐10 showed the highest NHC reduction capacity with DZP (4.17 ± 1.19) (Figure 4).

#### 3.1.4 Dark–Light Test

Figure 4 demonstrates that the MOR+DZP‐2 group's animals showed the lowest DRT (101.00 ± 9.85 s) in animals, which was then followed by DZP‐2, MOR‐10, and MOR‐5 by 119.33 ± 2.20, 124.20 ± 4.44 and 136.17 ± 4.02 s, respectively. All these groups significantly reduced DRT when compared to the control group data (143.33 ± 2.92 s). Nevertheless, FLU showed the highest DRT (145.83 ± 5.79 s) and the combination of FLU‐0.10 and MOR‐10 expressed a higher DRT (140.50 ± 5.46 s) than the DZP‐2 group. However, the FLU‐0.10 showed lower Cohen's D (Effect size) values compared to the control. The effect size in the open‐field test of all treatment groups is provided in Table S2.

### 3.2 *In Silico* Findings

#### 3.2.1 GABA_A_ Receptor Homology Modeling

**FIGURE 5:**
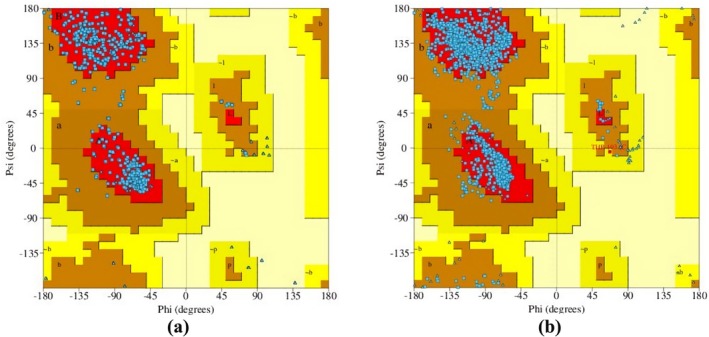
Ramachandran plot of the homology‐modeled GABA_A_ receptor: (a) α2 subunit; (b) α3 subunit.

The homology modeling results revealed that the target sequence of GABA_A_ receptor α2 and α3 subunits has sequence similarities of 57.18% and 76.50%, respectively, compared to the template sequence. The homology model of the human GABA_A_ receptor has been constructed with GMQE values of 0.60 and 0.66 for the α2 and α3 subunits, respectively. These values indicate that the constructed receptors are of good quality and reliability. The Ramachandran plot was assessed to verify the accuracy and reliability of the Psi and Phi angles of the residues. The plot displayed 92.70% and 91.90% of the most favored regions of the modeled receptors, along with 7.30% and 8.0% of regions that were additionally approved for the GABA_A_ receptor α2 and α3 subunits, respectively. The figure indicated that there were no disallowed regions for either receptor subunit, with a percentage of 0.00% (Figure 5).

#### 3.2.2 Interaction of Morellic Acid (MOR) and Diazepam (DZP) With GABA_A_ (α2 and α3) Receptor

From the *in silico* study, we found that MOR exhibited higher binding scores (−7.6 kcal/mol) towards the α2 subunit of the GABA_A_ receptor through the formation of four hydrogen bonds with the AA residues (A:ASP82, A:THR83, A:GLN217, and A:SER303) and two hydrophobic bonds: A:TYR252 (Pi‐Sigma) and A:TYR252 (Pi‐Alkyl) compared to the referral drug DZP, which showed −6.6 kcal/mol binding affinity towards the GABA_A_ α2 receptor by forming bonds with a number of AA residues: A:TYR252 (Pi‐Pi Stacked), A:GLY251 (Amide‐Pi Stacked), A:ILE255 (Alkyl), A:TYR252 (Pi‐Alkyl). However, FLU exerted the lowest binding affinity (−6.2 kcal/mol) towards the GABA_A_ α2 receptor through the formation of one hydrogen bond with the AA residue of ASP314 and several hydrophobic bonds (Alkyl and Pi‐alkyl) with the AA residues of ILE298, ARG301, ALA310, and ALA318.

**FIGURE 6:**
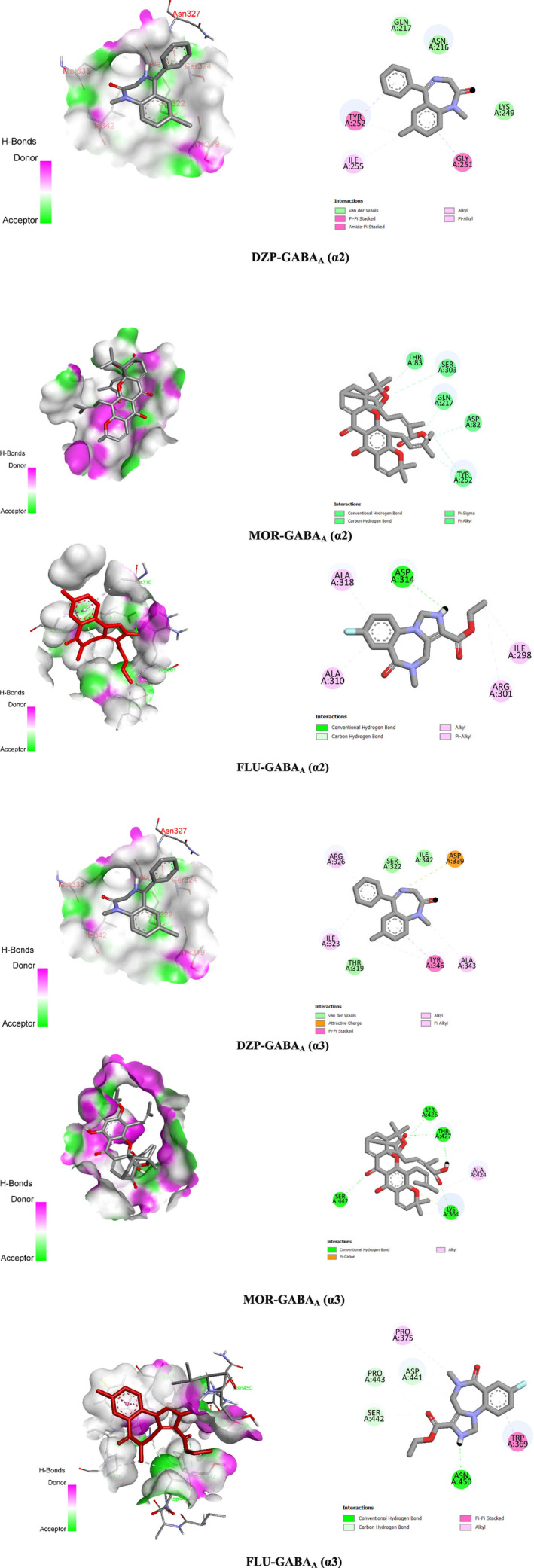
2D and 3D views of the receptor binding sites with the names of non‐bond interactions and amino acid residues of morellic acid, flumazenil, and diazepam‐GABA_A_ (α2 and α3) receptor complex.

On the other hand, MOR exerted the highest binding potential (−9.2 kcal/mol) towards the GABA_A_ α3 receptor and formed five hydrogen bonds (A:THR427, A:LYS364, A:SER426, A:THR427, and A:SER442) as well as several hydrophobic bonds with the AA chain: A:LYS364 (Pi‐Cation), A:ALA424 (Alkyl), and A:LYS364 (Alkyl). However, DZP disclosed a lower binding score (−7.3 kcal/mol) than MOR towards the α3 subunit of the GABA_A_ receptor by generating different hydrophobic bonds like A:ASP339 (Electrostatic bond), A:TYR346 (Pi‐Pi Stacked), (Alkyl), A:ALA343 (Alkyl), A:TYR346 (Pi‐Alkyl), and A:ILE323 (Pi‐Alkyl), A:ARG326 (Pi‐Alkyl). In contrast to MOR and DZP, FLU showed the lowest binding score (−6.3 kcal/mol) with the α3 subunit of the GABA_A_ receptor. Besides, FLU formed five hydrogen bonds, including A:ASN45 (1.96), A:SER442 (3.69), A:PRO44 (3.54), A:ASP441 (3.50), A:ASP441 (3.61) and two hydrophobic bonds such as A:TRP369 (Pi‐Pi Stacked) and A:PRO375 (Alkyl) while interacting with the GABA_A_ α3 receptor. These findings revealed that MOR showed greater binding affinity towards the α3 subunit of the GABA_A_ receptor, which leads to anxiolytic activity by reducing locomotor activity in mice. All the ligands demonstrated a reduced HB length below 3Ǻ (Table 2). 2D and 3D interactions and visualization are displayed in Figure 6.

**TABLE 2:** Best binding affinity results and non‐bond interactions of morellic acid and diazepam GABA_A_ (α2 and α3) receptor complex.

Macromolecule	Ligands	Binding affinity (kcal/mol)	No of HB	HB residues	HB distance (Å)	Other bonding residues
GABA_A_(α2)	DZP	−6.6	—	—	—	A:TYR252 (Pi‐Pi Stacked), A:GLY251 (Amide‐Pi Stacked), A:ILE255 (Alkyl), A:TYR252 (Pi‐Alkyl)
	MOR	−7.6	4	A:ASP82, A:THR83, A:GLN217, A:SER303	3.08, 2.20, 2.03, 3.61	A:TYR252 (Pi‐Sigma), A:TYR252 (Pi‐Alkyl)
	FLU	−6.2	1	A:ASP 314	3.09	A:ILE298 (Alkyl), A:ARG301 (Alkyl), A:ALA310 (Pi‐Alkyl), A:ALA318 (Pi‐Alkyl)
GABA_A_(α3)	DZP	−7.3	—	—	—	A:ASP339 (Electrostatic bond), A:TYR346 (Pi‐Pi Stacked), (Alkyl), A:ALA343 (Alkyl), A:TYR346 (Pi‐Alkyl), A:ILE323 (Pi‐Alkyl), A:ARG326 (Pi‐Alkyl)
	MOR	−9.2	5	A:THR427, A:LYS364, A:SER426, A:THR427, A:SER442	1.98, 2.12, 2.39, 2.37, 2.96	A:LYS364 (Pi‐Cation), A:ALA424 (Alkyl), A:LYS364 (Alkyl)
	FLU	−6.3	5	A:ASN450, A:SER442, A:PRO443, A:ASP441, A:ASP441	1.96, 3.69, 3.54, 3.50, 3.61	A:TRP369 (Pi‐Pi Stacked), A:PRO375 (Alkyl)

Abbreviations: DZP, Diazepam; FLU, Flumazenil; HB, hydrogen bond; MOR, Morellic acid.

#### 3.2.3 Pharmacokinetic and Drug‐Likeness Properties

Pharmacokinetic properties describe the attributes of a chemical's ADME (absorption, distribution, metabolism, and excretion). The drug development sector heavily depends on computational techniques derived from biological and chemical science. Various computational approaches, including SwissADME and the ProTox 3.0 server, can be employed to assess the ADMET properties of a chemical. “Drug‐likeness” refers to the method that demonstrates the process in which physical and chemical properties interact to regulate the formation of new drugs. Hydrogen bond donors (HBD), molar refractivity (MR), molecular weight (MW), hydrogen bond acceptor (HBA), and Log P are the major characteristics used to identify drug‐likeness. In this computational investigation, we found that the molecular weight of MOR is 560.63 g/mol, HBA of 8, and HBD of 2, MR of 154.06, and TPSA of 119.36 Å^2^. Findings also revealed that MOR is poorly soluble in water, and GI absorption is also very low. The compound follows all the rules to be a drug candidate, including Lipinski, Veber, and Egan, except Ghose and Muegge. However, one violation of Lipinski rules, one violation of Muegge rules, and three violations of Ghose rules are demonstrated. The bioavailability score of MOR is also demonstrated in this test, which is 0.56 (Table 3).

**TABLE 3:** Pharmacokinetic and drug‐likeness properties of morellic acid.

Parameters	Factors	Morellic acid
Physicochemical Properties	Formula	C_33_H_36_O_8_
	Molecular weight	560.63 g/mol
	Num. heavy atoms	41
	Num. arom. heavy atoms	6
	Fraction Csp3	0.48
	Num. rotatable bonds	5
	Num. H‐bond acceptors	8
	Num. H‐bond donors	2
	Molar Refractivity	154.06
	TPSA	119.36 Å^2^
**Lipophilicity**	Log P*o/w* (MLOGP)	2.45
**Solubility**	Water solubility	Poorly soluble
**Pharmacokinetics**	GI absorption	Low
	P‐gp substrate	Yes
	CYP1A2 inhibitor	No
	CYP2C19 inhibitor	No
	CYP3A4 inhibitor	Yes
	CYP2D6 inhibitor	No
	CYP2C9 inhibitor	Yes
	Log Kp (skin permeation)	−5.90 cm/s
**Druglikeness**	Lipinski	Yes; 1 violation: MW > 500
	Ghose	No; 3 violations: MW > 480, MR > 130, #atoms > 70
	Veber	Yes
	Egan	Yes
	Muegge	No; 1 violation: XLOGP3 > 5
	Bioavailability Score	0.56
**Medicinal Chemistry**	PAINS	0 alert
	Brenk	2 alerts: isolated_alkene, michael_acceptor_1
	Leadlikeness	No; 2 violations: MW > 350, XLOGP3 > 3.5
	Synthetic accessibility	7.00

### 3.3 Toxicity Analysis

**FIGURE 7:**
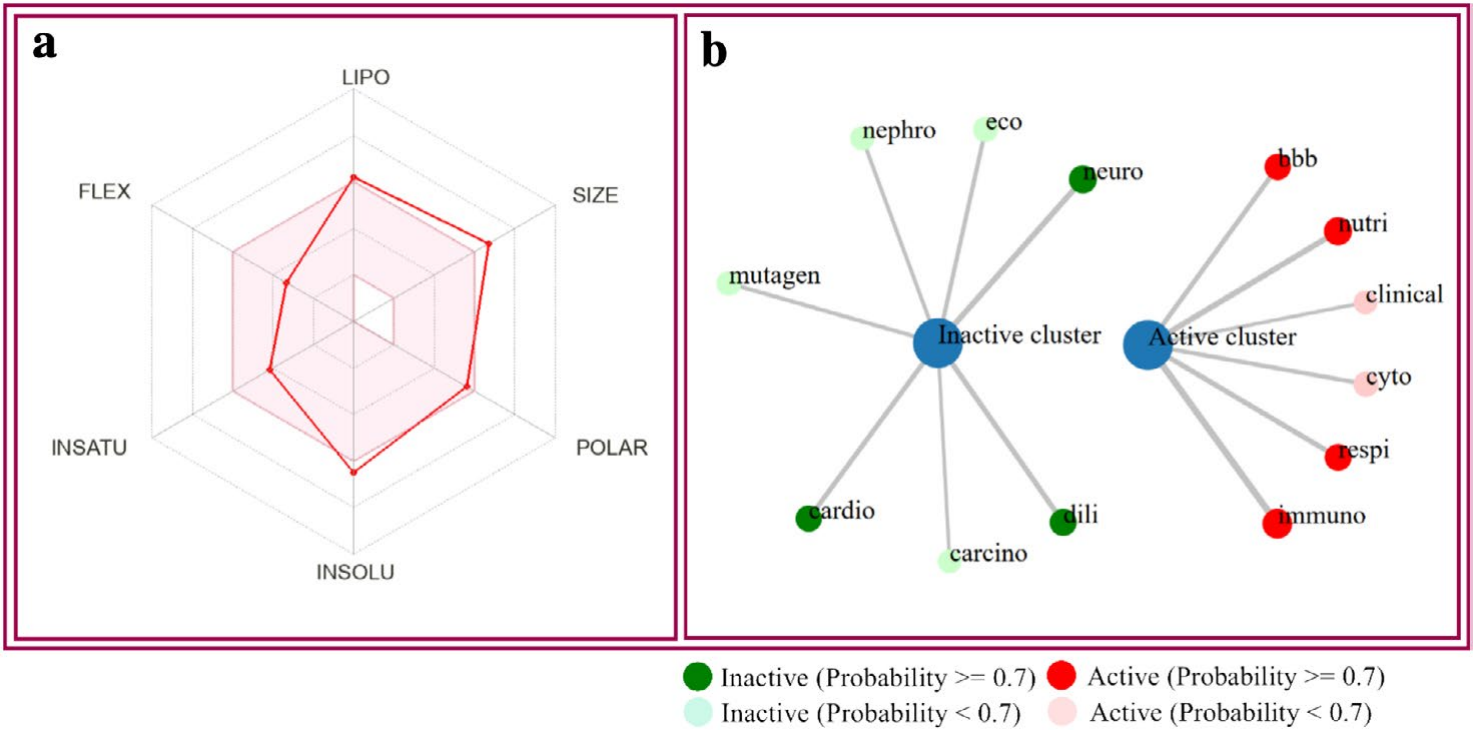
(a) Overview of the physicochemical, toxicological, and pharmacokinetic characteristics of morellic acid. The colored zone indicates the ideal physicochemical space for oral bioavailability. The parameters are defined as follows: SIZE: 150 g/mol<MV < 500 g/mol; INSOLU (Insolubility): −6 < log S (ESOL) < 0; LIPO (Lipophilicity): −7 < XLOGP3 < +5.0; INSATU (In saturation): 0.25 < Fraction Csp3 < 1; POLAR (Polarity): 20 Å^2^ < TPSA < 130 Å^2^; FLEX (Flexibility): 0 < num. rotatable bonds < 9. (b) The network chart is intended to quickly illustrate the connection between the selected compound (morellic acid and diazepam) and predicted activities. Abbreviation: bbb, blood brain barrier; carcino, carcinogenicity; cardio, cardiotoxicity; clinical, clinical toxicity; cyto, cytotoxicity; dili, drug‐induced liver injury; eco, ecotoxicity; immuno, immunotoxicity; mutagen, mutagenicity; nephro, nephrotoxicity; neuro, neurotoxicity; nutri, nutritional toxicity; respi, respiratory toxicity.

Our *in silico* toxicity investigation demonstrated that MOR exhibited immunotoxicity, respiratory toxicity, nutritional toxicity, cytotoxicity, and BBB‐barrier but did not show any other detectable harmful effects such as cardiotoxicity, carcinogenic properties, genetic toxicity, cell toxicity, or ecotoxicity. Moreover, the LD_50_ value for MOR was assessed to be 500 mg/kg, which reveals that MOR exhibited a significant safety margin, and its toxicity class is 4. According to Table 4, the ADMET properties of MOR were estimated using the SwissADME and ProTox 3.0 servers (Figure 7).

**TABLE 4:** An overview of the toxicity profile of MOR by using the ProTox 3.0 server.

Parameters	Report/Predicted value
	MOR
LD_50_	500 mg/kg
Toxicity class	4
Hepatotoxicity	Inactive
Neurotoxicity	Inactive
Nephrotoxicity	Inactive
Respiratory toxicity	Active
Cardiotoxicity	Inactive
Carcinogenicity	Inactive
Immunotoxicity	Active
Mutagenicity	Inactive
Cytotoxicity	Active
BBB‐barrier	Active
Ecotoxicity	Inactive
Clinical toxicity	Active
Nutritional toxicity	Active

Abbreviation: MOR, Morellic acid.

## 4 Discussion

This study revealed that MOR induces a calming and anxiolytic effect in *Swiss* albino mice, evidenced by a dose‐dependent decrease in locomotor activity, grooming, and rearing behaviors across multiple behavioral assessments. The OFT allows us to check the emotional state of the experimental animals [35, 36]. In the present study, both MOR and DZP remarkably reduced the NSC, NR, and NG parameters in the OFT. The reduction of NSC and NG parameters indicates that both DZP and MOR exerted a calming effect on the animals. On the other hand, the NR, or vertical movement, indicates a sign of locomotor activity, while the decreased number of horizontal movements indicates the central nervous system's (CNS) calming property of any test substance [37, 38]. MOR dose‐dependently decreased the horizontal movements of the animals, suggesting its calming effect on the CNS.

The hole‐board test allows us to check the anxiety behaviors of animals [39]. A decrease in the hole‐cross behavior (NHC) demonstrates a calming effect, that is, the anxiolytic property of the test substance [24]. In this study, we have seen that MOR dose‐dependently decreased the NHC in animals. On the other hand, the swing protocol was developed by Islam et al. [25] and aims to test the locomotor and CNS functions of experimental animals (e.g., mice and rats). The more movement of an animal inside the swing, the less the calming effect of a test substance [40]. Our study suggests that MOR dose‐dependently reduced NS, suggesting its calming behaviors. Finally, the dark–light protocol helps us to see the animals' tendency to avoid unfamiliar environments [41] by measuring the time spent (DRT) in the dark part of the chamber within the observation period [26]. An increase in DRT indicates the anxiolytic properties of the test substances. However, any test substance having a sedative‐anxiolytic‐like effect significantly decreased DRT in animals [42]. This study also indicated the elevated calming behavior of the experimental animals in MOR and DZP‐2 groups as all of the mice were kept in the light chamber and their movement was reduced, resulting in diminished time spent in the dark chamber [43]. In this study, we have seen that MOR dose‐dependently decreased DRT values in animals.

**FIGURE 8:**
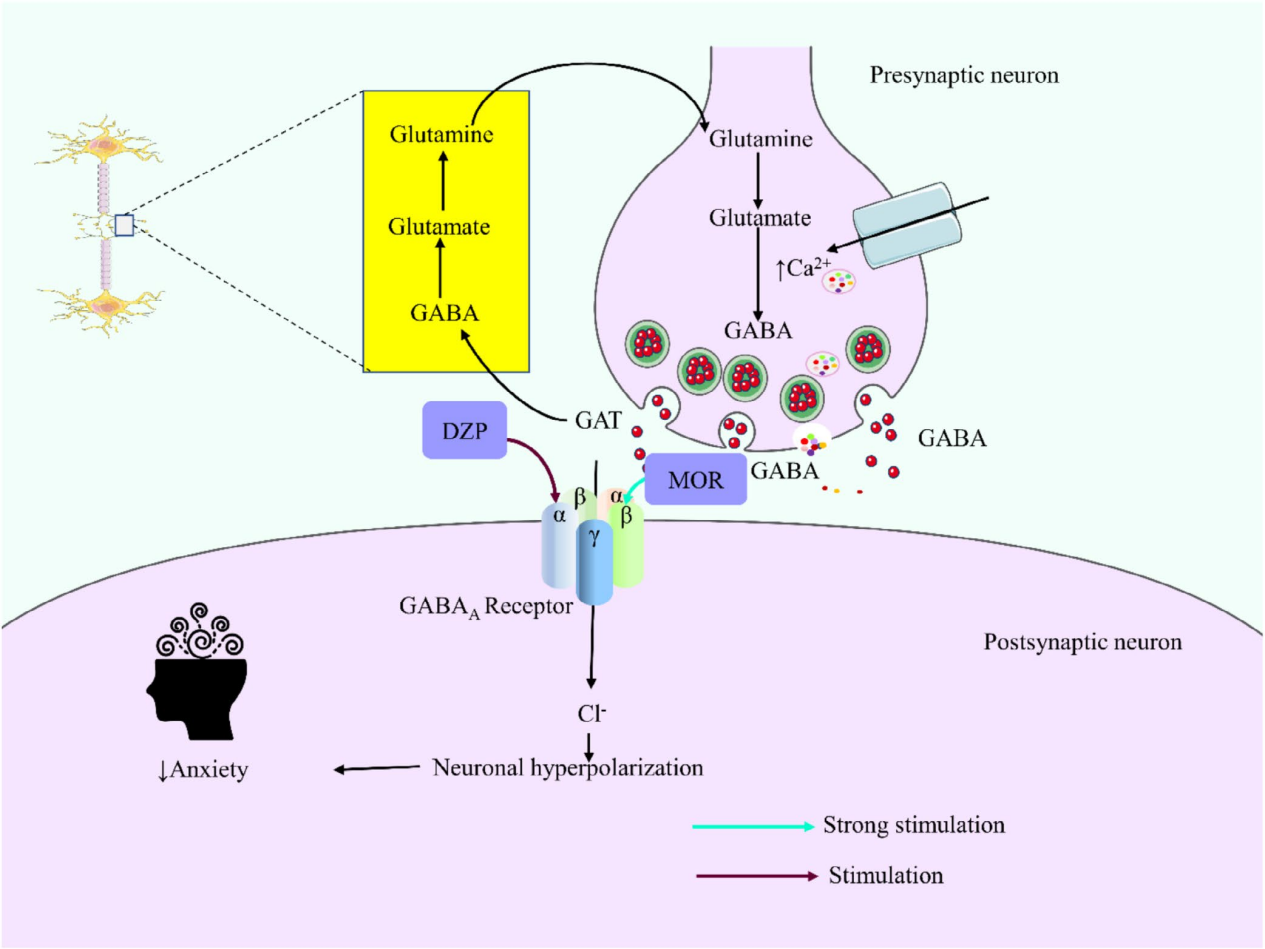
The possible anxiolytic mechanisms of morellic acid through GABA_A_ receptors. [Morellic acid (MOR) and diazepam (DZP) bind with the allosteric sites of GABA_A_ receptors and significantly hinder the Cl^−^ influx into the postsynaptic neuron, causing neuronal hyperpolarization resulting in anxiolytic activity].

From all these studies, it can be depicted that both the standard drug DZP and the substance MOR produced a calming effect on the animals. Therefore, our findings are in agreement with the earlier study, that is, the anti‐angiogenic effect of MOR on the zebrafish model [44]. In all cases, MOR combined with DZP augmented its effect in animals. It may have a synergistic effect with this sedative drug. GABA, an inhibitory neurotransmitter, regulates anxiety disorder [45]. Multiple animal studies have demonstrated that the transmission of GABA, a type of neurotransmitter, in the amygdala region of the brain has a substantial impact on the regulation of behavior related to anxiety [46]. The α subunits of the GABA_A_ receptor are responsible for its sensibility to GABA and also contribute to determining its pharmacological selectivity towards different allosteric modulators, such as DZPs and other drugs that interact with a similar site [47]. The principal inhibitory nerve cells in the central nervous system (CNS) that bind to benzodiazepines (BZDs) are the α subunits and γ subunits of GABA_A_ receptors (Olivier, Vinkers, & Olivier, 2013). DZP acts as a positive allosteric modulator of GABA_A_ receptors, causing the opening of chloride channels and an increase in chloride ion concentrations in neurons through its interaction with GABA [48]. This results in a hyperpolarization of the postsynaptic membranes, which enhances the negative response of the central nervous system to naturally existing GABA (Leão, Cabral, Izídio, Ribeiro, & Silva, 2016; [40] tfa). The experiment revealed that MOR had a strong anxiolytic impact on Swiss albino mice. This was seen in the dose‐dependent calming behaviors and reduction in locomotor activities. The data suggest that MOR could express anxiolytic effects that could be obtained by binding to GABA_A_ receptors and reducing locomotor activity, leading to a calming effect on the experimental subjects (Figure 8).

Molecular docking is a key method in molecular structural biology and computer‐aided drug discovery that is used to determine the potential binding affinity and attraction between ligands and receptors, as well as to assess the 2D and 3D structures of any chemical substance [49]. In this computer‐based study, we found that MOR exhibited significantly the highest binding affinity (−9.2 kcal/mol) towards the GABB_A_ α3 receptors and −7.6 kcal/mol with the GABB_A_ α2 receptor. These results represent that MOR has a remarkable role in anxiolytic disorder due to its higher binding affinity with both the GABB_A_ α2 and α3 receptors, which results in locomotor activity in test subjects and calming behavior. On the other hand, the referral drug DZP showed lower binding affinity, with a binding score of −6.6 and −7.3 kcal/mol towards the α2 and α3 subunits of the GABA_A_ receptor, respectively. Whereas FLU exhibited the lowest binding scores (−6.2 and −6.3 kcal/mol) with the GABA_A_ α2 and α3 receptors, respectively. The presence of a hydrogen bond is also an important factor because electron transfer between the ligand and receptor can improve the binding interaction of the ligand [50]. The ligand‐receptor visualization depicted that MOR formed four hydrogen bonds (A:ASP82, A:THR83, A:GLN217, and A:SER303) with the GABA_A_(α_2_) receptor and five hydrogen bonds (A:THR427, A:LYS364, A:SER426, A:THR427, and A:SER442) towards the GABA_A_(α_3_) receptor. In the case of the GABA_A_ α2 receptor, both the DZP and MOR demonstrated similar AA residues of A:TYR252, which indicated that these ligands have binding potential to the identical binding site, resulting in MOR exerting a DZP‐*like* effect.

From these findings, we suggested that MOR could exhibit anxiolytic activity by producing calming behavior and displaying reduced locomotor activity by interacting with the GABA_A_ receptor. It is possible that MOR could reduce the locomotor activity of mice by binding with the α3 subunit of the GABA_A_ receptor because it showed the highest binding scores with this subunit among the two subunits of the GABA_A_ receptor that are responsible for anxiolytic activity. However, only GABA_A_ receptor binding affinity cannot demonstrate the exact anxiolytic activity of MOR. Rather, it is also necessary to assess the levels of different anxiety‐related neurotransmitters, including DA, NE, and 5HT, in the synaptic cleft and postsynaptic neuron, as well as the transfer of the neurotransmitters in the presynaptic neuron.

Lipinski's rule of five mainly guides the development of novel therapeutics [51]. A chemical must follow specific criteria, including a MR within the range from 40 to 130, a molecular weight below 500 Da, a limited number of hydrogen bond donor and acceptor groups (less than 5 and 10, respectively), and a high level of lipophilicity (logP less than 5) [52]. According to SwissADME, MOR fulfills all the requirements and can thus be categorized as a drug‐like molecule.

There are some notable limitations in our study. Firstly, different external factors, including variations in the intensity of light, external noises, etc., could affect the in vivo test results. However, we tried our best to keep constant conditions for each subject. Secondly, some animals did not show any anxiety in uncomfortable or isolated conditions, which also affected the test results. Thirdly, we conducted the in vivo study over 2 days between 9 am and 3 pm, which could lightly affect the test results. Fourthly, gender is also an important factor in anxiety disorders, as females show greater anxiety‐related behaviors [53]. In this test, we used both male and female mice. Finally, there are different types of anxiety disorders in humans that can not be determined using animal model studies [54]. Our study does not demonstrate the exact anxiolytic process of MOR; rather, it suggests mechanisms only based on the anxiolytic effect of DZP and computational analysis.

## 5 Conclusion

MOR exerted a calming effect in a dose‐dependent manner in open‐field, hole‐cross, swing, and dark–light studies in *Swiss* albino mice. It also stimulated the calming effects of DZP in animals. *In silico* studies also suggest that MOR has a good binding capacity with −7.6 and −9.2 kcal/mol towards the α2 and α3 subunits of the GABA_A_ receptor, respectively. Both DZP and MOR had analogous AA residues of A:TYR252, suggesting that these ligands possess the potential to attach to the same binding site, hence causing MOR to provide a DZP‐like action. We suppose MOR may impart its anxiolytic effects in experimental animals, possibly by interacting with α2 and α3 subunits of the GABA_A_ receptor. MOR also revealed acceptable drug‐likeness and pharmacokinetics according to Lipinski's rule of 5. However, this study has substantial drawbacks. Firstly, its conclusion is based on behavioral observations in animals, which cannot precisely reflect the exact scenario inside the host's body. Secondly, the *in silico* investigations primarily focused on the GABA_A_ receptor, which may not be sufficient to determine the specific molecular mechanism behind the effect of MOR on anxiety. Additional research is highly recommended to validate these findings.

## Conflicts of Interest

The authors declare no conflicts of interest.

## Supporting information


**Table S1.** Cohen’s D (Effect size) value of different treatment groups in open field test.
**Table S2.** Cohen’s D (Effect size) value of different treatment groups in swing, hole cross, and dark–light tests.

## Data Availability

The data that support the findings of this study are available on request from the corresponding author. The data are not publicly available due to privacy or ethical restrictions.
