# Supplementary material for: Anxiolytic Activity of Morellic Acid: Modulation of Diazepam's Anxiolytic Effects, Possibly Through GABAergic Interventions
Source: CNS Neurosci Ther. 2025 Feb 17;31(2):e70276. doi: 10.1111/cns.70276 (PMC11831199; doi:10.1111/cns.70276)
Supplement: Supplementary file 1 — Table S1. Cohen’s D (Effect size) value of different treatment groups in open field test. Table S2. Cohen’s D (Effect size) value of different treatment groups in swing, hole cross, and dark–light tests. [file CNS-31-e70276-s001.docx]

**Supplementary**

**Anxiolytic activity of morellic acid: Modulation of diazepam’s anxiolytic effects, possibly through GABAergic interventions**

Md. Shimul Bhuia^1,2*^, Tanzila Akter Eity^2,3^, Raihan Chowdhury^1,2^, Siddique Akber Ansari^4^, Mehedi Hasan Bappi^2,5^, Md. Anin Nayeem^1,2^, Farjana Akter^2,6^, Muhammad Torequl Islam^1,2,7*^

*^1^Department of Pharmacy, Bangabandhu Sheikh Mujibur Rahman Science and Technology University, Gopalganj 8100, Bangladesh;* [*shimulbhuia.pharm@gmail.com*](mailto:shimulbhuia.pharm@gmail.com)*;* [*raihanpharmacy049@gmail.com*](mailto:raihanpharmacy049@gmail.com)*;* [*nayeem.phr@gmail.com*](mailto:nayeem.phr@gmail.com)

*^2^Bioinformatics and Drug Innovation Laboratory, BioLuster Research Center Ltd., Gopalganj 8100, Bangladesh*

*^3^Department of Biotechnology and Genetic Engineering, Bangabandhu Sheikh Mujibur Rahman Science and Technology University, Gopalganj 8100, Bangladesh;* [*tanzilaeity@gmail.com*](mailto:tanzilaeity@gmail.com)

*^4^Department of Pharmaceutical Chemistry, College of Pharmacy, King Saud University, P.O Box 2457, Riyadh 11451, Saudi Arabia;* [*sansari@ksu.edu.sa*](mailto:sansari@ksu.edu.sa)

*^5^School of Pharmacy, Jeonbuk National University, Jeonju-54896, Republic of Korea;* [*mehedibappi22@gmail.com*](mailto:mehedibappi22@gmail.com)

*^6^State University of Bangladesh, Purbacahal 1461, Dhaka, Bangladesh;* [*farjan.thesis@gnail.com*](mailto:farjan.thesis@gnail.com)

*^7^Pharmacy Discipline, Khulna University, Khulna 9208, Bangladesh*

**^*^Corresponding authors**: Md. Shimul Bhuia ([shimulbhuia.pharm@gmail.com](mailto:shimulbhuia.pharm@gmail.com)); Muhammad Torequl Islam ([dmt.islam@bsmrstu.edu.bd](mailto:dmt.islam@bsmrstu.edu.bd))

**Table S1.** Cohen’s D (Effect size) value of different treatment groups in open field test

| **Treatment groups** | **Cohen’s D (Effect size)** | | |
| --- | --- | --- | --- |
|  | **Square cross test** | **Grooming test** | **Rearing test** |
| Control (Vehicle) | - | - | - |
| DZP | 5.96 | 3.46 | 3.85 |
| FLU-0.10 | ‒1.48 | ‒0.49 | ‒0.38 |
| MOR-5 | 5.79 | 1.39 | 3.42 |
| MOR-10 | 7.09 | 1.73 | 4.02 |
| MOR-10+DZP | 7.46 | 3.49 | 3.71 |
| MOR-10+FLU-0.10 | 3.85 | 0.16 | 1.60 |
| DZP: Diazepam (2 mg/kg); FLU-0.10: Flumazenil (0.10 mg/kg); MOR-5 and 10: Morellic acid (5 and 10 mg/kg) | | | |

**Table S2.** Cohen’s D (Effect size) value of different treatment groups in swing, hole cross, and dark-light tests

| **Treatment groups** | **Cohen’s D (Effect size)** | | |
| --- | --- | --- | --- |
|  | **Swing test** | **Hole cross test** | **Dark-light test** |
| Control (Vehicle) | - | - | - |
| DZP | 3.32 | 2.90 | 3.39 |
| FLU-0.10 | ‒0.28 | ‒0.66 | ‒0.19 |
| MOR-5 | 2.59 | 2.26 | 0.74 |
| MOR-10 | 3.11 | 3.37 | 1.86 |
| MOR-10+DZP | 3.43 | 2.87 | 2.13 |
| MOR-10+FLU-0.10 | 1.69 | 1.05 | 0.24 |
| DZP: Diazepam (2 mg/kg); FLU-0.10: Flumazenil (0.10 mg/kg); MOR-5 and 10: Morellic acid (5 and 10 mg/kg) | | | |
